# Emodin *via* colonic irrigation modulates gut microbiota and reduces uremic toxins in rats with chronic kidney disease

**DOI:** 10.18632/oncotarget.8160

**Published:** 2016-03-17

**Authors:** Yu-Qun Zeng, Zhenhua Dai, Fuhua Lu, Zhaoyu Lu, Xusheng Liu, Cha Chen, Pinghua Qu, Dingcheng Li, Zhengshuang Hua, Yanni Qu, Chuan Zou

**Affiliations:** ^1^ Department of Nephrology, The Second Clinical College, Guangzhou University of Chinese Medicine and Guangdong Provincial Academy of Chinese Medical Sciences, Guangzhou, Guangdong, P.R. China; ^2^ Section of Immunology, The Second Clinical College, Guangzhou University of Chinese Medicine and Guangdong Provincial Academy of Chinese Medical Sciences, Guangzhou, Guangdong, P.R. China; ^3^ Department of Laboratory Medicine, The Second Clinical College, Guangzhou University of Chinese Medicine and Guangdong Provincial Academy of Chinese Medical Sciences, Guangzhou, Guangdong, P.R. China; ^4^ Biomedical Statistics and Informatics, Mayo Clinic, Rochester, MN, United States of America; ^5^ State Key Laboratory of Biocontrol, Key Laboratory of Biodiversity Dynamics and Conservation of Guangdong Higher Education Institutes, College of Ecology and Evolution, Sun Yat-sen University, Guangzhou, Guangdong, P.R. China

**Keywords:** emodin, colonic irrigation, gut microbiota, uremic toxins, chronic kidney disease, Pathology Section

## Abstract

Gut microbiota plays a dual role in chronic kidney disease (CKD) and is closely linked to production of uremic toxins. Strategies of reducing uremic toxins by targeting gut microbiota are emerging. It is known that Chinese medicine rhubarb enema can reduce uremic toxins and improve renal function. However, it remains unknown which ingredient or mechanism mediates its effect. Here we utilized a rat CKD model of 5/6 nephrectomy to evaluate the effect of emodin, a main ingredient of rhubarb, on gut microbiota and uremic toxins in CKD. Emodin was administered *via* colonic irrigation at 5ml (1mg/day) for four weeks. We found that emodin *via* colonic irrigation (ECI) altered levels of two important uremic toxins, urea and indoxyl sulfate (IS), and changed gut microbiota in rats with CKD. ECI remarkably reduced urea and IS and improved renal function. Pyrosequencing and Real-Time qPCR analyses revealed that ECI resumed the microbial balance from an abnormal status in CKD. We also demonstrated that ten genera were positively correlated with Urea while four genera exhibited the negative correlation. Moreover, three genera were positively correlated with IS. Therefore, emodin altered the gut microbiota structure. It reduced the number of harmful bacteria, such as *Clostridium spp.* that is positively correlated with both urea and IS, but augmented the number of beneficial bacteria, including *Lactobacillus spp.* that is negatively correlated with urea. Thus, changes in gut microbiota induced by emodin *via* colonic irrigation are closely associated with reduction in uremic toxins and mitigation of renal injury.

## INTRODUCTION

Chronic kidney diseases (CKD) have a complicated etiology and pathology and the mechanisms underlying their pathogenesis remain unclear. Uremic toxins promote progression of CKD with complications [1-3]. Mounting evidence has shown that uremic retention solutes originate from colonic microbial metabolisms, such as indoleamines derived from the bacterial metabolites, which can contribute to uremic toxins, including indoxyl sulfate (IS), through the gut-kidney axis [4, 5]. IS that originates from the gut microbiota fermentation turns out to be an independent risky factor for CKD and cardiovascular diseases (CVD) [6]. On the other hand, previous studies have suggested that gut microbiota are altered under CKD state and that this dysbiosis in turn contributes to the progression of CKD [7, 8]. Recent studies have revealed some effective treatments of the CKD with altering uremic toxins and gut microbiota through targeting the colonic environment, including oral administration of AST-120 to adsorb uremic toxins [9], and prebiotics or probiotics to modulate gut microbiota [8, 10]. However, the relevance of gut microbiota to CKD and uremic toxins is not well understood.

Emodin (1,3,8-trihydroxy-6-methylanthraquinone) is a naturally occurring anthraquinone present in the roots and barks of numerous plants [11, 12], and especially, an important active ingredient of Chinese herb Rhubarb (Da Huang) [13]. Emodin is laxative, antibacterial, immunosuppressive, and diuretic [14-17]. It is often used for the treatment of constipation. Previous researches have also shown that emodin glycoside is cleaved by the intestinal bacteria to release emodin, which in turn activates the underlying smooth muscle cells, leading to muscle contraction. It also inhibits the activity of K_ATP_ channel and the ion transport (chloride) across colon cells [18] due to its laxative effect. Rhubarb, a laxative Chinese medical herb since ancient time, has been widely used to reduce uremic toxins for the treatment of CKD in China [19, 20]. But it has been used more often *via* colonic administration than via oral administration since early 1950s [21]. Our previous studies have demonstrated that rhubarb-based decoction enema reduces the uremic toxins, including urea, creatinine and IS, preserves the renal function and help patients avoid the dialysis [22]. But mechanisms underlying its effects are not understood. We speculated that treatments with rhubarb *via* colonic route modulate the multiple targets on gut microbiota and metabolic toxins through the gut-kidney axis to improve the renal function. Here, we examined structural alterations of gut microbiota and levels of uremic toxins in response to emodin colonic irrigation (ECI). We found that ECI markedly reduced the levels of uremic toxins, including urea and IS, and modulated the gut microbiota. In particular, ECI reduced the number of harmful bacteria, such as *Clostridium spp.* but increased the number of beneficial bacteria, including *Lactobacillus spp.*

## RESULTS

### Kidney function is improved by ECI

Data are summarized in Table 1. Compared with the sham-operation control group (CTL), CKD groups with either emodin colonic irrigation (ECI) or control colonic irrigation (CCI) showed a significant increase in creatinine and urea concentrations as well as urinary protein excretion, but an decrease in hemoglobin and hematocrit levels, creatinine clearance and body weight. Then compared with CCI control groups, ECI significantly lowered urea concentrations and urinary protein excretion, slightly reduced creatinine without statistic significance, improved creatinine clearance and body weight, and increased hemoglobin and hematocrit levels.

**Table 1:** Changes in body weight and renal function after ECI or CCI

	BW(g)	Creatinine(mg/dl)	Urea (mg/dl)	Hb(g/L)	U PCR(g/g Cr)	Hematocrit(%)	Ccr(ml/min/kg)
CTL	389±14.15	0.40±0.06	48±13.51	145±7.20	0.54±0.11	40.8±2.28	6..09±1.15
CCI	350±13.00*	0.97±0.12*	114±25.80*	127±10.75*	16.24±4.02*	35.7±2.55*	2.45±1.23*
ECI	359±13.10*	0.92±0.10*	84±12.40^Δ^	129±16.18*	9.48±2.34^Δ^	36.1±5.39*	3.21±0.29*

Body weights were measured and serum samples were collected and analyzed 4 weeks after CCI or ECI treatments following the 5/6 nephrectomy.

Abbreviations: BW, body weight; Ccr, creatinine clearance rate; Hb, hemoglobin;

U PCR, unrine protein creatinine ratio; Cr, serum creatinine; CTL, sham-operation control without 5/6 nephrectomy; ECI, emodin colonic irrigation; CCI, control colonic irrigation.

Values are presented as Mean ± SD

* *p* < 0.05 compared with CTL;

Δ *p* < 0.05 compared with CCI.

### ECI significantly reduced uremic toxin IS

**Figure 1:**
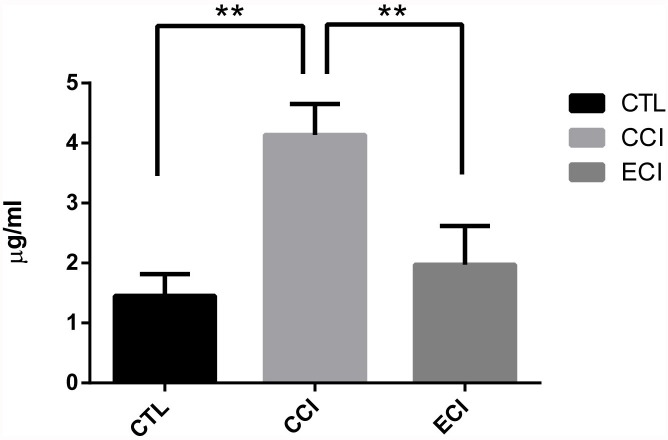
Concentrations of the indoxyl sulfate (IS) in serum after ECI or CCI (μg/ml) Serum samples were collected and analyzed by HPLC-MS 4 weeks after CCI or ECI treatments following the 5/6 nephrectomy. Data are presented as Mean ± SD *p < 0.05, **p < 0.01.

After the treatments for 4 weeks, the serum samples were analyzed by HPLC-MS, as shown in Figure 1. When compared with the control (CTL), experimental groups with 5/6 nephrectomy and CCI control exhibited a significant increase in the IS levels (4.13±0.52 *vs* 1.45±0.36 μg/mL; *p* < 0.01). However, ECI treatments significantly reduced its level compared with CCI control (1.97±0.64 *vs* 4.13±0.52 μg/mL; *p* < 0.01). There was no statistical difference between CTL controls and ECI groups.

### The dominant fecal microbiota changes with ECI

**Figure 2:**
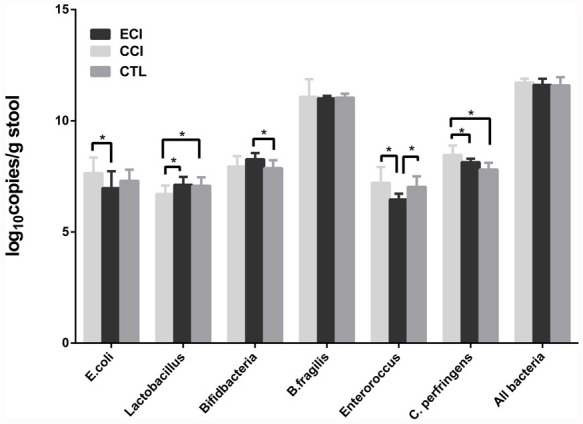
Quantifications of bacteria in the fecal microbiota (log_10_copies/g stool) Real-time qPCR was performed to detect the fecal microbiota 4 weeks after CCI or ECI treatments following the 5/6 nephrectomy. Data are presented as Mean ± SD, *p < 0.05.

The dominant fecal microbiota were profiled by real-time qPCR. There was no significant change with total numbers of all bacteria among all groups. Compared with the CTL group, *Clostridium Perfringens* (*C. perfringens*) in experimental CCI control group was increased significantly, whereas the *Lactobacillus* was significantly decreased. Moreover, compared with the CCI control group, *Lactobacillus* in ECI group was remarkably increased whereas *Enteroroccus*, *Escherichia coli* (*E. coli*) and *C. perfringens* in ECI group was significantly reduced. Finally, *Bacteroides fragilis* (*B. fragilis*) did not change among all groups (Table 2 and Figure 2).

**Table 2:** Quantification of bacteria in the fecal microbiota by Real-time qPCR (log10copies/g stool)

	E.coli	Lactobacillus	Bifidbacteria	B.fragilis	Enteroroccus	C. perfringens	All bacteria
CTL	7.30±0.50	7.08±0.38	7.87±0.36	11.04±0.18	7.03±0.47	7.81±0.30	11.59±0.37
CCI	7.65±0.70	6.71±0.38*	7.95±0.46	11.08±0.79	7.21±0.71	8.46±0.43*	11.72±0.18
ECI	6.97±0.76^Δ^	7.12±0.36^Δ^	8.27±0.28*	11.01±0.11	6.46±0.26^Δ^	8.14±0.16^Δ^	11.61±0.28

* *P* < 0.05, compared with CTL.

Δ *P* < 0.05, compared with CCI

### Overall structural changes of gut microbiota after ECI treatments

**Figure 3:**
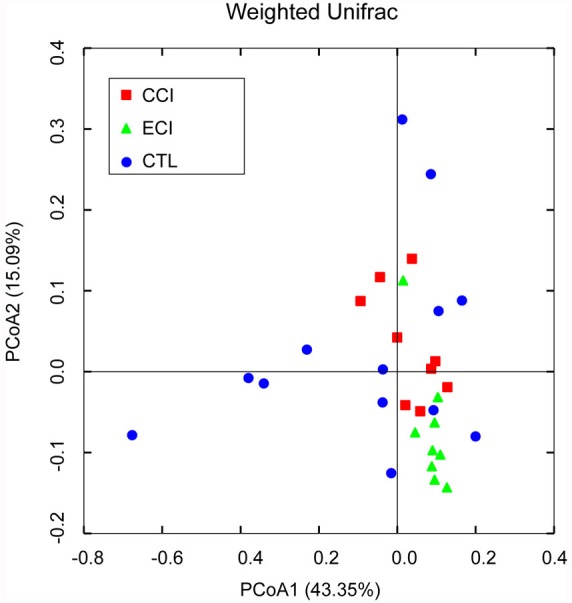
Weighted Unifrac PCoA analysis of gut microbiota based on the OTU data from pyrosequencing run A point represents a sample from each group. The sample numbers (n) in each group: CTL = 13, CCI = 9, and ECI = 9.

Using a bar-coded pyrosequencing of V3-V5 region of 16S rRNA genes of 128,238 valid reads in the three groups from 31 samples, we got total 3,744 operational taxonomic units (OTUs) with an average of 488 OTUs per sample (±120 sd.). The Venn diagram determined the shared and unique OTUs that some different structures were showed in each group (Supplementary Figure S1). The rarefaction curves and the alpha diversity analysis have shown the most characters of the samples, with most diversities being captured (Supplementary Figure S2 and Table S1). Weighted and unweighted UniFrac PCoA analysis revealed that the gut microbiota structure of the three groups exhibited a deviation away from one another (Figure 3).

### Key microbiota changes following ECI and its association with uremic toxins including urea and IS

**Figure 4:**
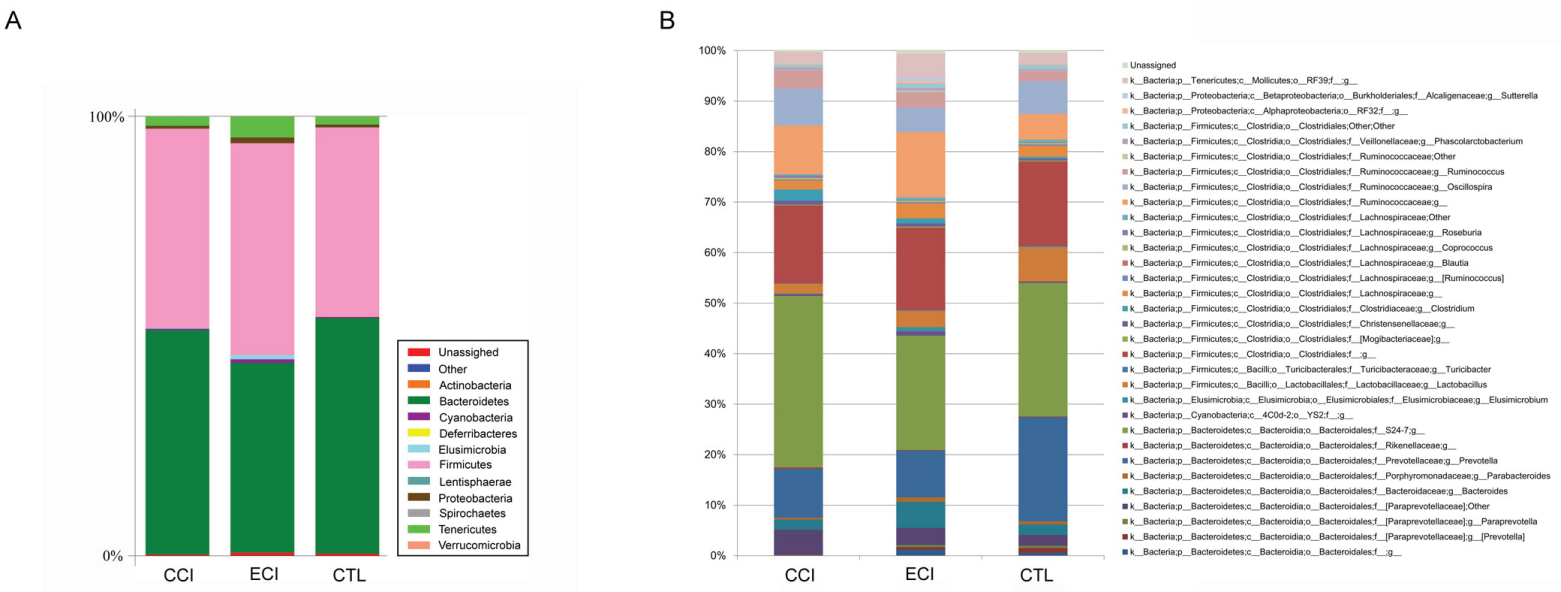
Relative abundance of the gut microbiota **A.** abundance and prevalence of the different bacterial phyla in each group. **B.** abundance and prevalence of the different bacterial genera in each group.

In this study, we provided a broad view of the gut microbiota. A total of 8 phyla, 12 classes, 13 orders, 32 families, and 47 genera were represented. At the phylum level, the most abundant were *Bacteroidetes* (51.1% counts in CCI control group, 43.2% in ECI group and 53.7% counts in CTL control group), *Firmicutes* (45.4%counts in CCI control group, 48.3% in ECI group and 43.1%counts in CTL control group), *Tenericutes* (2.0%counts in CCI control group, 4.7% in ECI group and 1.8%counts in CTL control group), and *Proteobacteria* (0.7%counts in CIC control group, 1.3% in ECI group and 0.7%counts in CTL control group) (Figure 4A). The four phyla were the predominant ones constituting 98.6% of total microbiota.

As described in other studies [7, 23, 24], *Firmicutes*, *Bacteroidetes* and *Proteobacteria* phyla were consistently present with the greatest numbers. But in our study, *Tenericutes* was also very abundant and significantly increased in ECI group compared with CCI control or CTL control group (*P* < 0.05), while there was no difference in other three phyla between all groups. The phyla of *Actinobacteria* and *Spirochaetes* were only found in ECI group with 0.1% counts for both. The remaining bacteria belonged to the *Cyanobacteria*, *Deferribacteres*, *Elusimicrobia*, *Lentisphaerae*, *Spirochaetes* and *Verrucomicrobia* with less than 0.5% counts.

At the genus level, there was a difference in 47 genera (Figure 4B), with 3 genera found only in CTL group, 6 genera only in CCI control group, and 3 genera only in ECI group. Among all genera, the genera from the families of *Prevotellaceae*, *S24-7*, *Bacteroidaceae*, *Paraprevotellaceae*, *Lactobacillaceae*, *Ruminococcaceaeha*, *Lachnospiraceae* and unclassified *Clostridiales* order were dominant. The five classified genera (*Bacteroides*, *Prevotella*, *Lactobacillus*, *Oscillospira*, and *Ruminococcus)* were most abundant in each group. Meanwhile, compared with the CTL, *Lactobacillus* was decreased significantly in CCI control group while genera of *Ruminococcus*, *Clostridium* and unclassified genus from *Ruminococcaceae* family, which were all from *Clostridiales* order, were increased in CCI control group. Then compared with CCI control group, the unclassified genera from *S24-7* family and *Clostridium* were remarkably decreased in ECI group, whereas *Bacteroides* and unclassified genus from *RF39* order were markedly increased in ECI group with a slight improvement of the *Lactobacillus* also in ECI group without statistical significance. Among all genera observed, only *Adlercreutzia*, *Bacteroides*, unclassified genera from *RF39* order and *Ruminococcaceae* family were significantly increased in ECI group compared with CTL control group.

**Figure 5:**
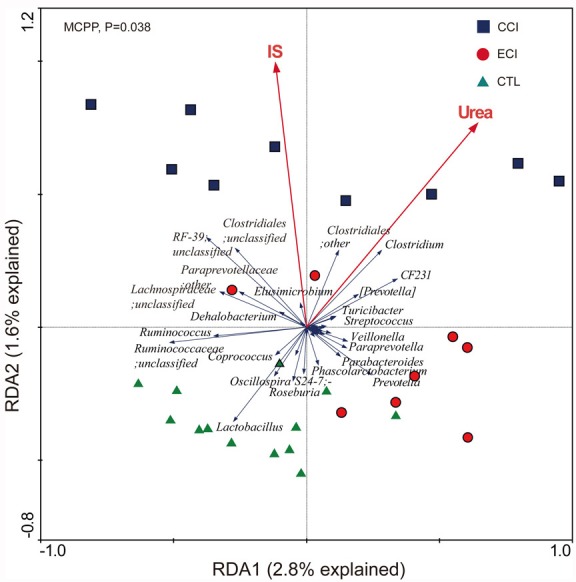
Biplot of redundancy analysis (RDA) of the gut microbiota compositions after ECI treatments in CKD rats The uremic toxins of Urea and IS were used as environmental variables. On Top-left, *P*-value was obtained by Monte Carlo permutation procedure (MCPP).

**Figure 6:**
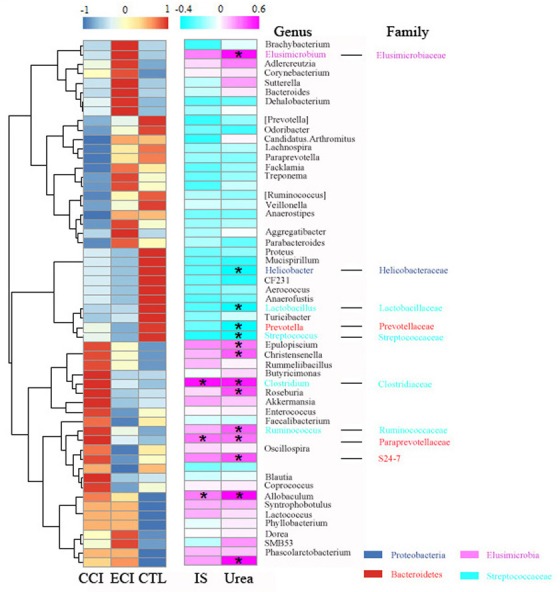
Heatmap of key bacterial genera responding to ECI treatments and Spearman's correlation between genera and Urea or IS The color of spots in the left panel represents the relative abundance of the genus in each group. The color of spots in the right panel represents R-value of Spearman's correlation between genera and Urea or IS. The family and genus names are shown on the right. *p < 0.05.

Using redundancy analyses, we chose 56 core genera that responded to ECI treatments (Figure 5). We found that 10 genera (unclassified *S24-7* family, unclassified *Paraprevotellaceaea* family, *Ruminococcus*, *Clostridium*, *Elusimicrobium*, *Roseburia*, *Allobaculum*, *Epulopiscium*, and *Christensenella*) exhibited a significant positive correlation with Urea, while 4 genera (*Prevotella*, *Lactobacillus*, *Streptococcus*, and *Helicobacter*) had a significant negative correlation with Urea. 3 genera (unclassified *Paraprevotellaceae* family, *Clostridium* and *Allobaculum*) showed a significant positive correlation with IS, but no genus had a significant negative correlation with IS. The genus of *Streptococcus* trended to have a negative correlation with IS (Figure 6). *Bifidobacterium* genus was undetectable in all groups.

## DISCUSSION

In this study, we sought to understand the mechanisms by which ECI improves renal function by focusing on the change of gut microbiota resulted from ECI intervention since gut microbiota has been shown to play a role in the generation of uremic toxins [4, 5]. We found that ECI markedly reduced the levels of uremic toxins, including urea and IS, and modulated the gut microbiota.

Uremia is largely due to the accumulation of organic waste products. At present, a treatment of uremia is to slow the progression of kidney failure or to finally replace kidney function with dialysis or a transplant. But, it has been difficult to reduce uremic toxins using western medicines. Unfortunately, dialysis causes many complications in CKD patients and lowers their quality of life. Kidney transplantation is also limited due to insufficient donors. Recently emerging are new approaches to treating uremia and preserving kidney function using Chinese medicine enema. For instance, rhubarb enema is one of the most important Chinese medicines for the treatment of various diseases. It is widely used to clear away uremic toxins and improve the uremic symptoms in China [21]. The current study demonstrated that ECI reduced the accumulation of uremic toxins urea and IS, which was consistent with our previous clinical observation [22]. Moreover, it also remarkably reduced the urinary protein excretion, suggesting that ECI slows the progression of kidney failure.

As a uremic toxin, IS is derived from gut microbial metabolism [25] and associated with progression of CKD and mortality [26]. When proteins are not digested in the gastrointestinal tract, fermentation of the amino acid tryptophan by intestinal microbiota generates indoles, which are further hydroxylated into 3-hydroxy-indole to generate IS. The latter circulates in equilibrium between a free solute fraction and a fraction bound to serum proteins to damage kidney function [5]. Urea, another important uremic toxin, is also associated with colonic environment where it is hydrolyzed by microbial urease to form ammonia [CO(NH_2_)2+H_2_O→CO_2_+2NH_3_], and it is a likely mediator that alters gut microbiota [27, 28]. These two uremic toxins damage intestinal barrier, leading to systemic inflammation with injured kidney functions [29]. The decrease in these uremic toxins and changes of gut microbiota after ECI treatments in this study suggest that gut microbiota is correlated with the uremic toxins.

Gut microbiota, mainly residing in the colon as a forgotten “big organ” [30], has approximately 500 different bacterial species and 10^14^ cells that is 10 times of total human cells, and plays an important role in human health and diseases [31]. The microbiota is complex, diverse, and stable within a big individual for long-term [32, 33]. But it changes with outer environment [34] and is a closely linked to CKD [8]. Vaziri et al have shown a significant difference in the abundance of 175 bacterial OTUs between uremic rats with 5/6 nephrectomy and control animals, with a significant decrease in the *Lactobacillaceae* and *Prevotellaceae* families in rats with 5/6 nephrectomy [7], similar to the findings by Mishima et al showing that abundance of *Lactobacillaceae* and *Prevotellaceae* families was decreased in adenine induced CKD mice [35]. We also found that the *Lactobacillus* genus from *Lactobacillaceae* family was markedly reduced in CCI control group of CRF according to both pyrosequencing and real-time PCR analyses. Like studies by Hide et al [36], the total number of bacteria in our study exhibited no any significant difference between each group, while the number of *C. perfringens* was increased in CCI control group with CRF. We also found that genera of *Ruminococcus*, *Clostridium* and unclassified genus from *Ruminococcaceae* family were also increased in CCI control group. A recent study demonstrated significant improvements in renal function as well as colonic inflammation and epithelial barrier structure in CKD rats fed with a high fiber diet [37], suggesting an important impact of the dietary restriction on the structure and function of microbiome. Thus, it remains to be defined how ECI treatments with dietary restriction would alter the gut microbiota.

Among strategies of improving renal function in CKD by reducing uremic toxins, new therapies targeting the colonic microenvironment aim to modulate gut microbiota or clear uremic toxin solutes derived from the microbiota metabolisms. We first detected the rat fecal microbiota and found that *Clostridium* was remarkably decreased after ECI treatments. *Lactobacillus* was improved in ECI group, while *C. perfringens* was reduced. But we found that *Enteroroccus* was markedly reduced while *Bifidbacteria* was improved with ECI treatments according to real-time quantitative PCR, though barely detectable by pyrosequencing. Hide et al reported that *Enteroroccus* was augmented but *Bifidbacteria* was decreased in CKD status, suggesting that the gut microbiota tends to resume the original balance in the healthy status upon ECI treatments.

Bacterial species are roughly categorized as the saccharolytic or proteolytic. Wong et al used *Silico* tests to find that bacterial families possessing urease, uricase, and indole-and p-cresol-forming enzymes were expanded while butyrate producing bacteria were reduced in patients with ESRD [38]. Similar to the findings by Wong et al, the harmful genus of *Clostridium* from *Clostridiaceae* family possessing urease and indole-forming enzymes was increased in CCI control with CRF and positively correlated with urea and IS, but this increase was offset by ECI treatments. In particular, the increase in *C. perfringens* from *Clostridium* genus was also abrogated by ECI treatments. Some studies also showed that *Clostridium difficile* from *Clostridium* genus is associated with increased mortality for CKD and ESRD [39, 40]. This finding proved that *Clostridium* is a bad bacterial genus, which increases the production of urea and IS in CKD, and that ECI treatments may reverse the course. On the other hand, the bacterial genera of *Lactobacillus* and *Prevotella* from the *Lactobacillaceae* and *Prevotellaceae* families possessing butyrate-producing enzymes were reduced in CCI control with CRF and negatively correlated with urea, while ECI treatments significantly rescued the genus of *Lactobacillus*, but not *Prevotella*. These two genera have been reported to confer beneficial effects of improving butyrate that can expand regulatory T cells in the large intestine [41]. Therefore, these ECI-induced beneficial genera may help attenuate inflammation by suppressing pro-inflammatory cells in CKD. It is noteworthy that the association of uremic toxins with gut microbiota changes induced by ECI is not in a cause-effect manner. Future studies are needed for establishing such a manner.

## MATERIALS AND METHODS

### Animals, interventions and samples

Male Sprague-Dawley rats with a body weight of approximately 200g were used and purchased from Experimental Animal Center of Guangdong Province (Guangzhou, China). Animals were housed in a specific pathogen free (SPF) environment with 12 hours of light-dark cycles and were fed with standard laboratory food and water ad libitum. The animals were randomly assigned to the sham-operation control groups (CTL) and experimental groups. All experimental CRF groups underwent 5/6 of nephrectomy *via* surgical removal of the upper and lower thirds of the left kidney, and resection of whole right kidney seven days later. The procedures were conducted under common anesthesia using strictly aseptic techniques. The animals with CRF again were randomly assigned to the emodin colonic irrigation (ECI) and control colonic irrigation (CCI) groups 8 weeks after 5/6 of nephrectomy. Animals of ECI groups were given emodin *via* colonic irrigation using a lavage needle (8 cm) through the anus, at 5 mL(1 mg/day) for four weeks, and the water containing 0.4% CMC-Na was used to dissolve emodin for ECI treatments or to serve as CCI control group without emodin. Four weeks after the irrigation, fresh fecal, urine and serum samples were collected from the animal trails. All the samples were stored at −80°C until analyses. All animal experiments in this study were approved by the Institutional Animal Care and Use Committee of the Second Clinical College, Guangzhou University of Chinese Medicine(Guangzhou, China), and were performed in accordance with the Declaration of National Institutes of Health Guide for the Care and Use of Laboratory Animals (Publication No. 80-23, 1996).

### Serum preparation and quantitative detection of uremic toxin indoxyl sulfate (IS)

The chemicals, including the standard IS potassium salt (purity 99.8%), were obtained from Sigma-Aldrich (St. Louis, MO, USA), and the internal standard of hydrochlorothiazide (purity 99.5%) was obtained from Institute for Drug Control (Guangzhou, China), while methanol (LC-MS grade) and ammonium acetate were purchased from Sigma-Aldrich.

Firstly, 100 μL of fixed serum was pipetted into 1.5 mL polypropylene tube, and 500 μL hydrochlorothiazide in methanol solution (25 μg/mL) was added to precipitate the protein. The tubes were mixed by vortex for 1 minute. After centrifugation at 12,000 rpm for 10 minutes, 100 μL clear supernatant was extracted and mixed with 100 μL ddH_2_O to filter into a new polypropylene tube, and transferred to an auto-sampler. The curve standards were prepared at 0.25, 0.5, l, 5, 10, 50 μg/mL in methanol for IS, dried out using vacuum spinner (Thermo, Waltham, MA, USA), re-dissolved with normal rats' serum, and finally analyzed as described in the serum method. Low, medium and high concentrations of quality-control (QC) samples were also prepared. The IS-quantitative method was described in our previous studies [42].

### Fecal bacterial DNA extraction

Each fecal bacterial DNA was extracted using the QIAamp DNA Stool Mini kit (QIAGEN, GmbH, Germany) according to the manufacturer's protocols. The completed DNA samples were stored at −80°C before use.

### Analysis of dominant fecal microbiota by Real-Time quantitative PCR

Quantification of gene expression was performed *via* the LightCycler System I (BioRad, Richmond, CA, USA) using iQ^Tm^ SYBR Green SuperMix (BioRad). Reaction mixtures (20 μL) contained 10 μL of 2 × SYBR-Mix, 1 μL of template DNA, 1 μL of each primer, and 7 μL of ddH2O. The primers for 5′ nuclease assays were based on sequences of the 16S rRNA region of special species, as listed in Supplementary Table S3. Real-time PCR quantification of species-specific DNA was performed using a standard curve generated from the dilution series (from 1×10^2^ to 1×10^9^ copies/μL) of a standard strain (Supplementary Table S4). Standard and quantified samples were run in triplicates. The temperature program for the qPCR included an initial denaturation step at 95°C for 3min followed by 40 cycles of amplification at 95°C for 10 s, annealing at 60°C for 30 s, and elongation at 72°C for 60 s. The melting curve was obtained by gradual heating with a 0.5°C/s increment from 50°C to 95°C and continuous fluorescence measurements. The results were expressed as 16S rRNA gene copies per wet weight of feces.

### Pyrosequencing

The V3-V5 regions in the 16S rRNA gene of Escherichia coli were amplified by PCR from extracted DNA using sample-unique DNA bar-coded primers (forward: 5′-CCGTCAA- TTCMTTTGAGTTT-3′, reverse: 5′-ACTCCTACGGGAGGCAGCAG-3′) [43]. The PCR reaction program was as follows: 3 min initial denaturation at 94°C, 20 cycles of denaturation at 94°C for 1min, annealing from 65°C to 55°C (1°C reduction for every two cycles) for 1 min, elongation at 72°C for 1 min, and the final extension at 72°C for 6 min [44]. Then, the resulting products were verified *via* gel electrophoresis. DNA pyrosequencing was carried out on 454 GS FLX+ platform (Roche, Branford, CT, USA).

### Bioinformatics and statistical analyses of sequencing data

Raw data generated from the 454-pyrosequencing running were processed following the pipelines of Mothur (version 1.29) [45] and QIIME according to Kuang's method [46]. Briefly, the low quality sequences were trimmed with an average quality score less than 20 using a 50 bp sliding window, and chimeric sequences were identified and removed using UCHIME with *de novo* method [47]. Quality sequences were subsequently assigned to samples according to their unique 10-bp barcode and binned into phylotypes using average clustering algorithm at the 97% similarity level. Representative sequences were aligned using NAST [48] and taxonomic classification of phylotypes was obtained using the Ribosomal Database Project (RDP) classifier (at least 80% threshold) [49]. In addition, the phylogenetic tree of representative sequences of operational taxonomic units (OTUs) was built using FastTree [50] with default parameters. Finally OTU table was tabulated by combining the information of relative abundance in each sample and taxonomic classification for each OTU.

The α- and β- diversities were performed using QIIME [51]. The established phylogenetic tree and the relative abundance table of representative sequences of OTUs were used for Weighted UniFrac analysis and UniFrac principal coordinate analysis (PCoA) [52]. We obtained the abundance differences in microbial communities from three groups, and FDR (false discovery rate) was adopted to assess the significance of differences using one-way ANOVA (*p* < 0.05). We identified the urea and IS as environmental variables using Redundancy analysis with CANOCO, and the statistical significance was assessed by MCPP. The Spearman's correlation coefficient (R) and P value were used to evaluate the relationship between the urea, IS and pyrosequencing. All statistical analyses were performed using various packages with the R statistical computing environment.

### Statistical analyses

Values were expressed as means ± standard deviations. One-way ANOVA and Student-Newman-Kueuls Q tests were used for comparison analyses of continuous variables using GraphPad Prism 5.0 (GraphPad Software, La Jolla, CA). A probability level of < 0.05 was considered statistically significant.

## SUPPLEMENTARY MATERIAL TABLES AND FIGURES


