# Supplementary material for: Emodin via colonic irrigation modulates gut microbiota and reduces uremic toxins in rats with chronic kidney disease
Source: Oncotarget. 2016 Mar 17;7(14):17468–78. doi: 10.18632/oncotarget.8160 (PMC4951226; doi:10.18632/oncotarget.8160)
Supplement: Supplementary file 1 [file oncotarget-07-17468-s001.pdf]

Emodin *via* colonic irrigation modulates gut microbiota and reduces uremic toxins in rats with chronic kidney disease

Supplementary Material

Supplementary Table S1 Alpha diversity analyses of samples

Sample ID	Chao1	Simpson	Shannon	OTUs	PD[a]
CTL01	1346	0.97	6.8	459	46
CTL02	1516	0.98	7.4	531	55
CTL03	458	0.97	6.3	247	28
CTL04	1026	0.92	6.3	419	44
CTL05	1361	0.97	7.3	494	53
CTL06	907	0.85	5.5	359	39
CTL07	1116	0.86	5.8	428	45
CTL08	1131	0.97	6.8	409	41
CTL09	965	0.97	6.8	419	43
CTL10	1249	0.97	6.7	406	41
CTL11	793	0.97	6.8	350	39
CTL12	793	0.97	6.6	356	36
CTL13	511	0.71	4.0	244	31
CCI01	1342	0.98	7.1	457	48
CCI02	405	0.96	6.0	237	28
CCI03	472	0.98	6.7	259	29
CCI04	433	0.97	6.2	240	28
CCI05	1316	0.99	7.5	478	47
CCI06	1564	0.98	7.3	509	49
CCI07	477	0.97	6.5	252	28
CCI08	470	0.98	6.8	281	31
CCI09	1322	0.97	7.0	482	50
ECI01	1540	0.98	7.0	455	47
ECI02	1997	0.99	7.9	622	60
ECI03	1114	0.98	7.3	459	48
ECI04	993	0.98	7.3	485	50
ECI05	1806	0.99	7.7	575	57
ECI06	1532	0.98	7.6	547	56
ECI07	1407	0.98	7.1	465	46
ECI08	1313	0.98	7.1	465	48
ECI09	1711	0.98	7.4	558	55

a. PD is an abbreviation for phylogenetic diversity.

Supplementary Table S2 Mass transitions and compound parameters

	Q1（m/z）	Q3（m/z）	DP	FP	EP	CE	CXP
IS	212	81	-25	-150	-4	-33	-10
hydrochlorothiazide	296	269	-120	-200	-10	-27	-6

DP, declustering potential; FP, potential; EP, entrance potential; CE, collision energy; CXP, collision exit potential.

Supplementary Table S3 Primers and products used in the real-time qPCR

Targer bacteria	Primer	Sequence(5'-3')	Product size(bp)	Reference
Escherichia coli	Forward	CATGCCGCGTGTATGAAGAA	95	1
	Reverse	CGGGTAACGTCAATGAGCAAA		
Lactobacillus spp.	Forward	TGGATGCCTTGGCAC TAGGA	92	2
	Reverse	AAATCTCCGGATCAAAGCTTACTTAT		
Bifidbacteria spp.	Forward	GCGTGCTTAACACATGCAAGTC	124	3
	Reverse	CACCCGTTTCCAGGAGCTATT		
Bacterodides.fragilis	Forward	GAGAGGAAGGTCCCCCAC	106	4
	Reverse	CGCTACTTGGCTGGTTCAG		
Enteroroccus spp.	Forward	CCCTTATTGTTAGTTGCCATCATT	144	5
	Reverse	ACTCGTTGTACTTCCCATTGT		
Clostridium perfringens	Forward	ATGCAAGTCGAGCGATG	120	5
	Reverse	TATGCGGTATTAATCTTCCTTT		
All bacteria	Forward	TCCTACGGGAGGCAGCAGT	466	6
	Reverse	GGACTACCAGGGTATCTAATCCTGTT		

Table S4 Standard bacterial strains in this study

Organism	strain[a]
Escherichia coli	ATCC25922
Bifidobacterium longum	ATCC15707
Enterococcus faecalis	ATCC29212
Lactobacillus bulgaricus	ACCC10638
Clostridium perfringens	ATCC13124
Bacteroides fragilis	ATCC25283

[a] ATCC,American Type Culture Collection；ACCC, Agricultural Culture Collection of China

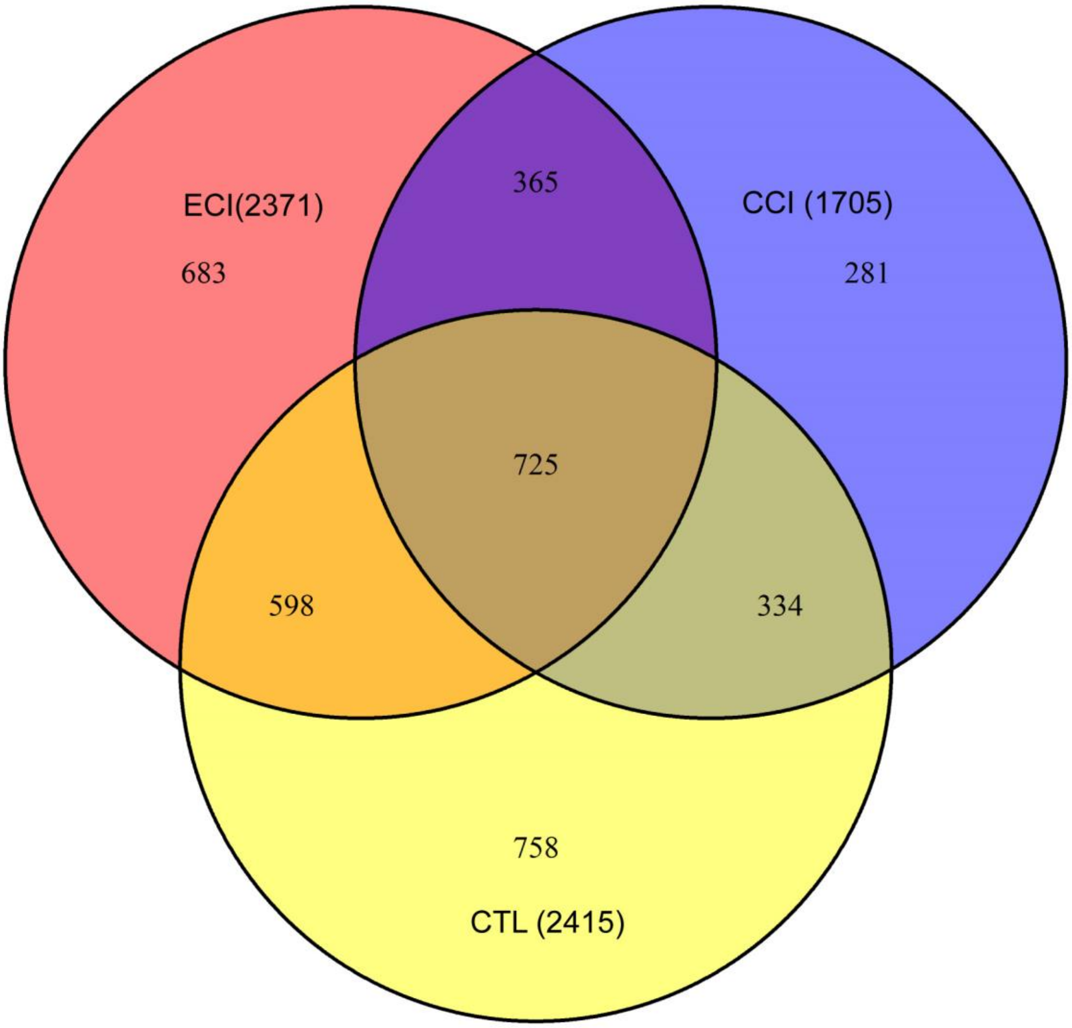

Supplementary Figure S1 Venn diagrams of numbers of OTUs common or unique to each group.

Each circle represents a group. The interior of each circle represents the number of OTUs in each group. The overlapping areas represent the set of OTUs commonly present in some counterpart groups. The single-layer zone represents the number of OTUs specifically found in a particular group.

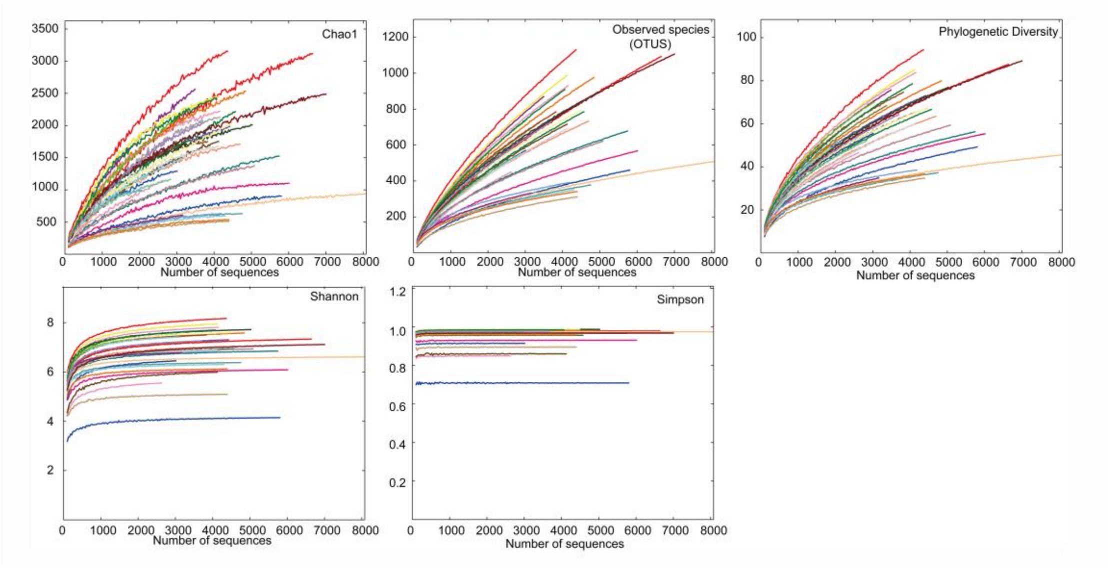

Supplementary Figure S2 Rarefaction curves. Rarefaction measurements of the gut microbiota with Chao1, Observed species (OTUs), phylogenetic diversity, Shannon and Simpson show the diversities of OTUs in each sample.

References

1. Huijsdens XW, Linskens RK, Mak M, Meuwissen SG *et al*. Quantification of bacteria adherent to gastrointestinal mucosa by real-time PCR. *J Clin Microbiol*. **40**, 4423-4427(2002).

2. Haarman M, Knol J. Quantitative real-time PCR analysis of fecal Lactobacillus species in infants receiving a prebiotic infant formula. *Appl Environ Microbiol*. **72**, 2359-2365(2006).

3. Penders J, Vink C, Driessen C, London N *et al*. Quantification of Bifidobacterium spp., Escherichia coli and Clostridium difficile in faecal samples of breast-fed and formula-fed infants by real-time PCR. *Fems Microbiol Lett*. **243**, 141-147(2005).

4. Layton A, McKay L, Williams D, Garrett V *et al*. Development of Bacteroides 16S rRNA gene TaqMan-based real-time PCR assays for estimation of total, human, and bovine fecal pollution in water. *Appl Environ Microbiol*. **72**, 4214-4224(2006).

5. Rinttila T, Kassinen A, Malinen E, Krogius L *et al*. Development of an extensive set of 16S rDNA-targeted primers for quantification of pathogenic and indigenous bacteria in faecal samples by real-time PCR. *J Appl Microbiol*. **97**, 1166-1177(2004).

6. Nadkarni MA, Martin FE, Jacques NA, Hunter N. Determination of bacterial load by real-time PCR using a broad-range (universal) probe and primers set. *Microbiology+*. **148**, 257-266(2002).
